# Study and Application of Acoustic Emission Testing in Fault Diagnosis of Low-Speed Heavy-Duty Gears

**DOI:** 10.3390/s110100599

**Published:** 2011-01-10

**Authors:** Lixin Gao, Fenlou Zai, Shanbin Su, Huaqing Wang, Peng Chen, Limei Liu

**Affiliations:** 1 Key Laboratory of Advanced Manufacturing Technology, Beijing University of Technology, Chao Yang District, Beijing, 100124, China; E-Mail: lead0003@163.com (L.X.G.); 2 School of Mechanical & Electrical Engineering, Beijing University of Chemical Technology, Chao Yang District, Beijing, 100029, China; 3 Graduate School of Bioresources, Mie University, 1577 Kurimamachiya-cho, Tsu, Mie, 514-8507, Japan; E-Mail: chen@bio.mie-u.ac.jp; 4 Tang Shan Iron & Steel CO. LTD., Tangshan, Hebei, China

**Keywords:** acoustic emission, low-speed and heavy-duty, gear fault diagnosis, redundant second generation wavelet

## Abstract

Most present studies on the acoustic emission signals of rotating machinery are experiment-oriented, while few of them involve on-spot applications. In this study, a method of redundant second generation wavelet transform based on the principle of interpolated subdivision was developed. With this method, subdivision was not needed during the decomposition. The lengths of approximation signals and detail signals were the same as those of original ones, so the data volume was twice that of original signals; besides, the data redundancy characteristic also guaranteed the excellent analysis effect of the method. The analysis of the acoustic emission data from the faults of on-spot low-speed heavy-duty gears validated the redundant second generation wavelet transform in the processing and denoising of acoustic emission signals. Furthermore, the analysis illustrated that the acoustic emission testing could be used in the fault diagnosis of on-spot low-speed heavy-duty gears and could be a significant supplement to vibration testing diagnosis.

## 1. Introduction

A gear system is a power and motion transmission device that is applied most extensively in various kinds of industrial equipment. Its operational state directly affects the function of the whole equipment. Faults and failures of gears can cause great damage to the whole production. Therefore, the diagnosis of gear faults is of significant importance.

The background noise in the fault signals of low-speed heavy-duty gears is complicated and of low energy, so conventional vibration testing methods are not effective. The acoustic emission is the high-frequency stress-wave signal emitted due to structural imperfections. Compared with vibration signals, the frequency spectra of acoustic emission signals are broader, and their high frequencies can inhibit the noise interferences effectively and improve the diagnosis accuracy. Besides, any dynamic imperfections can be detected through acoustic emission, and it is unnecessary to force the detected piece to approach the detection device. Therefore, there are great advantages in predicting and diagnosing the faults of low-speed heavy-duty gears with acoustic emission. It is worth noting that most previous studies have been focused on the analysis of laboratory data. On the one hand, they make the necessary preparations for the on-spot application of acoustic emission testing; on the other hand, noises interfere with the on-spot testing of acoustic emission. The application of acoustic emission to the fault diagnosis of low-speed heavy-duty gears is presently a research hot spot and of great practical significance to the industrial production management.

## 2. Principles of Acoustic Emission Testing

**Figure 1.**
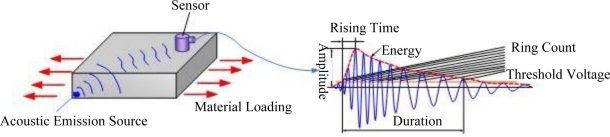
Principle of Acoustic Emission Testing.

Acoustic emission testing refers to a technique of testing, recording and analyzing acoustic emission signals using apparatus as well as speculating on the status of an acoustic emission source as normal or not based on acoustic emission signals. The principle of acoustic emission testing is shown in Figure 1: the elastic waves sent from the acoustic emission source are transmitted to the material surface via a transmission media and converted to electric signals by sensors before being magnified, processed and recorded. Through the analysis and processing of acquired signals, any defects inside the material could be detected.

As for the methods of processing the acoustic emission signals of rotating machinery, presently there are parameter analysis methods and waveform analysis methods. The former ones are dominated by methods based on basic parameters such as the ring, energy and amplitudes [1]; compared with the original waveforms of signals, however, these parameters lose massive information and have difficulties in characterizing the essence of defects. Technicians at home and abroad have done a lot of research on waveform analysis methods. By building a theoretical model, Bashir simulated the acoustic emission energy which was sent upon the extension of the fine cracks on the rolling bearings in a helicopter gearcase. The extension course of cracks could be tested in real time, and the bearing faults could be detected before surface materials were peeled off bearings [2]. McFadden employed acoustic emission sensors to test the signals of angular contact thrust bearings under low-speed rotation. He noticed that, in low-speed rotation, the acoustic emission sensors could detect the acoustic emission signals induced by the concentrated load of rolling elements [3]. Mba *et al*. distinguished the types of bearing faults using the acoustic emission technique and auto-regressive coefficients. They obtained substantial results, but did not validate them in practice [4–8].

In recent years, wavelet technology has been widely applied to the testing and diagnosis of heavy-duty equipment in China and other countries. Based on the general framework of morphological undecimated wavelets, Zhang *et al*. employed the morphological opening operation and the multi-scale Top-Hat transform as the analysis operators for the approximation signals and the detail signals in wavelet decomposition, respectively. Through tests, they verified the feasibility and validity of the method [9]. He *et al*. used wavelet scalograms to analyze in detail the time-frequency, propagation and dispersion characteristics of rubbing acoustic emission [10]. Deng *et al*. extracted the high-frequency components containing the fault signals of spindles for envelopment analysis and detected successfully fault frequencies [11]. Classical wavelet construction generally depends on the Fourier transform in the frequency domain. After the construction, the wavelet shape is fixed and difficult to match waveforms with different signal characteristic. In order to overcome the above drawbacks, Sweldens put forward a promotion algorithm for the construction of wavelet functions, which was known as the second generation wavelet transform [12]. With promotion steps and corresponding principles being applied to the design of predictors and updaters, wavelets with expected characteristics can be constructed and applied in the fault diagnosis of mechanical equipment. Li *et al*. analyzed the drawbacks of the promotion algorithm and the redundant promotion algorithm. Aiming at the cause for the generation of error transfer in the redundant promotion algorithm, they put forward an improved redundant promotion algorithm based on normalizing factors and extracted successfully the characteristics of faint fault signals using the shock pulse method [13].

Zhao eliminated the background noise of acoustic emission signals through wavelet analysis and reconstruction, and then identified the faults of rolling bearings using the wavelet envelopment spectrum analysis method. Test results proved that the faults of rolling bearings could be detected effectively with the wavelet envelopment spectrum analysis [14]. Using the wavelet packet technique, Yao extracted the characteristics of the acoustic emission signals during the crack extension on bearings and identified acoustic emission sources through soft demodulation [15].

The above research achievements show that, although acoustic emission signals are one of a few effective carriers that can be acquired from the testing diagnosis information of low-speed heavy-duty equipment, there are still great difficulties in identifying the early faults in such equipment by using acoustic emission signals. So far, the research has basically been limited to the laboratory stage, and inadequate studies have made about the applicability of on-spot engineering. The low-speed heavy-duty equipment in real operation bears enormous alternating load and various kinds of shock. With the motions and mechanical frictions, *etc*. of water, oil, gas and other types of liquid being taken into account, there are abundant signals and one of the acknowledged puzzles has been how to identify early faults effectively in the presence of strong background noise.

In this study, a redundant second generation wavelet transform method was constructed and it was validated that the redundant second generation wavelet transform was effective in the processing and denoising of acoustic emission signals through the analysis of the acoustic emission data from the faults of on-spot low-speed heavy-duty gears.

## 3. Study on Redundant Second Generation Wavelet Algorithm

### 3.1. Redundant Second Generation Wavelet Construction Algorithm

The redundant second generation wavelet transform includes two processes: decomposition and reconstruction. The decomposition includes two parts: prediction and renovation. The reconstruction includes the reconstruction recovery and the renovation recovery. During the decomposition and reconstruction, the length of a signal sequence remains fixed. Symbols *P* and *U* in the algorithm represent the predictor and the updater, respectively.

The decomposition is as follows: the original signal sequence is denoted by *s*(*n*), with the data length as *L*. The course of the redundant second generation wavelet decomposition is expressed as follows:

1. Prediction. Each sample in the signal sequence is predicted with adjacent 2*^l^* *N* samples through predictor *P*, and prediction error {*d*_*l*+1_ (*n*), *n* ∈ *Z*} is defined as detail signals:
  (1) $$\begin{array}{l} d_{l+1}\;(k)=s_{l}\;(k)-\\ \left[p_{1}s_{l}\;(k-2^{l-1}(N-1))+p_{2}s_{l}\;(k-2^{l-1}(N-3))+\cdots +p_{N}s_{l}\;(k+2^{l-1}(N-1)\right] \end{array}$$
2. Renovation. Based on detail signal *d*_*l*+1_ (*n*), updater *U* is used to renovate each sample in the signal sequence using 2*^l^* *N* detail signals. The signal sequence *s*_*l*+1_ (*n*) obtained after the renovation is defined as approximation signals:
  (2) $$\begin{array}{l} s_{l+1}\;(k)=s_{l}\;(k)+\\ \left[u_{1}d_{l+1}\;(k-2^{l-1}(N-1))+u_{2}d_{l+1}\;(k-2^{l-1}(N-3))+\cdots +u_{N}d_{l+1}\;(k+2^{l-1}(N-1))\right] \end{array}$$

The reconstruction course is as follows:

1. Renovation recovery. Sample sequence $s_{l}^{u}$ is recovered with approximation signal *s*_*l*+1_ and detail signal *d*_*l*+1_:
  (3) $$\begin{array}{l} s_{l}^{u}\;(k)=s_{l+1}\;(k)-\\ \left[u_{1}d_{l+1}\;(k-2^{l-1}(N-1))+u_{2}d_{l+1}\;(k-2^{l-1}(N-3))+\cdots +u_{N}d_{l+1}\;(k+2^{l-1}(N-1))\right] \end{array}$$
2. Prediction recovery. Sample sequence $s_{l}^{p}$ is recovered with approximation signal $s_{l}^{u}$ and detail signal $s_{l}^{u}$:
  (4) $$\begin{array}{l} s_{l}^{p}(k)=d_{l+1}(k)+\\ \left[p_{1}s_{l}^{u}(k-2^{l-1}(N-1))+p_{2}s_{l}^{u}(k-2^{l-1}(N-3))+\cdots +p_{N}s_{l}^{u}(k+2^{l-1}(N-1))\right] \end{array}$$
3. Sample sequence $s_{l}^{u}$ after the renovation recovery and sample sequence $s_{l}^{p}$ after the prediction recovery are averaged, and the result is used as reconstruction signal *s_l_*:
  (5) $$s_{l}(n)=\frac{1}{2}[s_{l}^{u}(n)+s_{l}^{p}(n)]$$

### 3.2. Construction Method of Predictor and Updater

**Figure 2.**
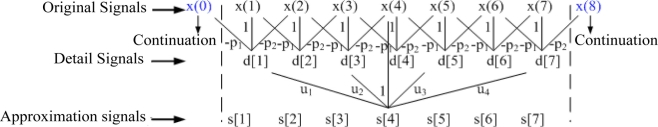
The decomposition process of redundant second generation wavelet.

**Figure 3.**
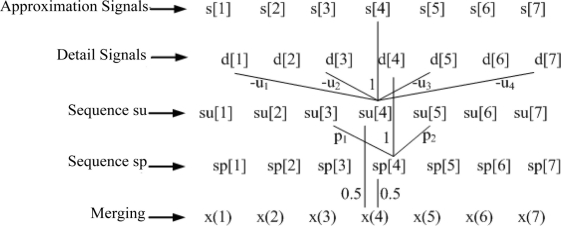
The reconstruction process of redundant second generation wavelet.

The updater length can be different from the predictor length, e.g., *Ñ* = 8. In order to calculate the updater coefficient at *Ñ* = 8, the predictor coefficient at *N* = 8 needs to be calculated first, then a half of the predictor coefficient is the result. Figures 2 and 3 show the decomposition and reconstruction processes at the predictor length of 2 and the updater length of 4.

### 3.3. Comparison between the Denoising Effect of Redundant Second Generation Wavelet and General Wavelet

**Figure 4.**
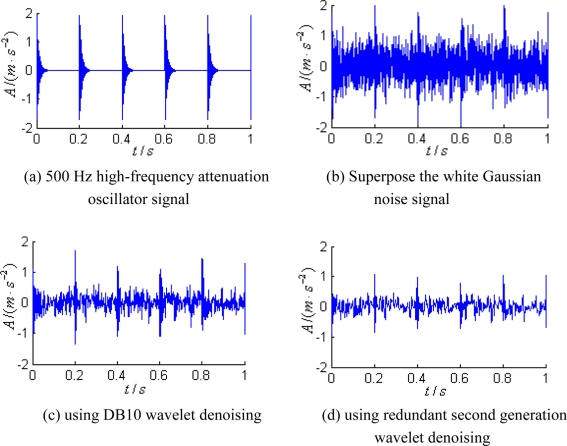
Comparison illustrations for signal denoising.

We construct a high-frequency attenuation oscillator signal with the frequency of 500 Hz, as shown in Figure 4(a). White Gaussian noise is superposed in the signal, as shown in Figure 4(b). The traditional wavelet and the redundant second generation wavelet are used to conduct the denoising on signals. DB10 wavelet is selected as the traditional wavelet for the 3-layer wavelet decomposition, while soft threshold and adaptive noise reduction are chosen as the denoise threshold, the denoising results as shown in Figure 4(c); the signals were decomposed with redundant second generation wavelet into three-layer and the lengths of the predictor and the updater were set at 8, soft threshold denoising and self-adaptive denoising were employed, and the empirical value *c* was set to 2.9, the denoising results as shown in Figure 4(d).

The energy ratio and standard deviation of signals were calculated using the two wavelets denoising. The energy ratio and standard deviation of signal using DB10 wavelet denoising are 0.5012, 16.2809; the energy ratio and standard deviation of signal using redundant second generation wavelet denoising are 0.3755, 18.2142. From the denoising results, it can be seen that the redundant second generation wavelet has de-noised more noise signals than traditional wavelet; from the comparison between energy ratio and standard deviation, it can be seen that the redundant second generation wavelet is more ideal.

## 4. Analysis of Acoustic Emission Data from Faults of On-Spot Gears Based on Redundant Second Generation Wavelet

The acoustic emission testing was conducted on seven rough bar mills in a particular steel plant on 23 June 2010; the employed testing apparatus was a SAEU2S acoustic emission scanner produced by the Beijing Shenghua Xingye Technological Co. Ltd.

**Figure 5.**
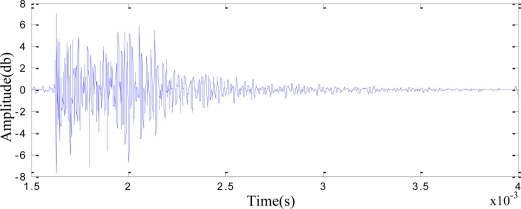
AE signals of lead-breaking in time domain.

Before beginning the signal acquisition, we needed to conduct sensitivity calibration for the sensors. The signals generated by the pencil lead-breaking is very similar to the signals produced by the metal crack propagation, therefore, we can use the pencil lead as a source of simulated AE signals to conduct detection. The collected signal is as shown in Figure 5. From Figure 5, it can be clearly seen that burst-type acoustic emission signal is generated by pencil lead break, which indicates that the sensitivity of the sensor has met the requirements, so the data acquisition is feasible.

**Figure 6.**
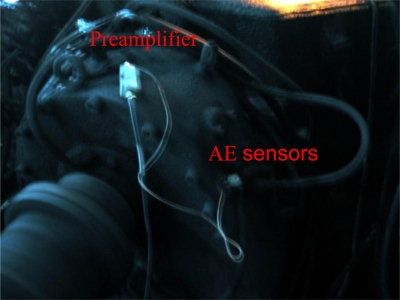
Installation site of sensors.

The installation site for sensors was shown in Figure 6. Set the sampling frequency to 800 KHz and the sampling site number to 262144.

**Figure 7.**
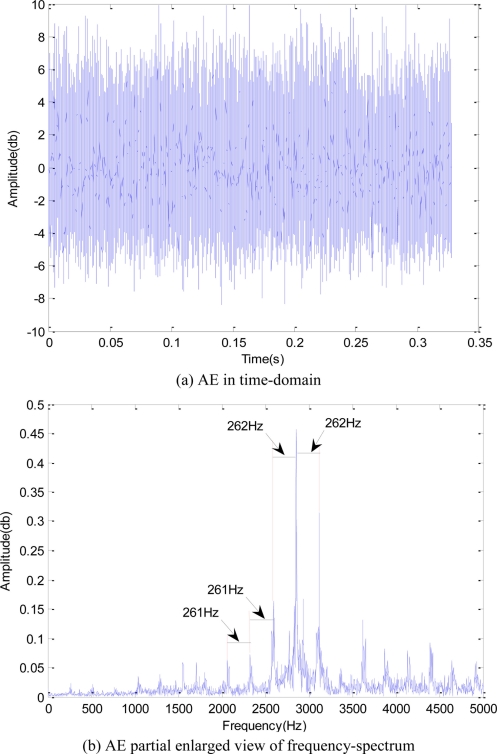
AE signals in time-domain and frequency-domain.

Figure 7 shows the time-domain and the frequency-domain of the acoustic emission. From the time-domain figure, no significant cyclic shock component could be seen; from the original frequency-spectrum figure, harmonics with intervals about 261 Hz could be seen. The AE signals were denoised with redundant second generation wavelet; afterwards, the Hilbert demodulation was performed.

The procedure for redundant second generation wavelet denoising being performed on signals is the following:

Step 1: Determine the number of signal decomposition layers of redundant second generation wavelet; in this paper, the AE signals were decomposed with redundant second generation wavelet into four-layer and the lengths of the predictor and the updater were set at 20;
Step 2: Determine the threshold; in this paper, soft threshold denoising and self-adaptive denoising were employed. The empirical value *c* was set to 2.9.
Step 3: Reconstruct the details signal processed with the threshold and approximation signal, and then the denoised signal is obtained.

**Figure 8.**
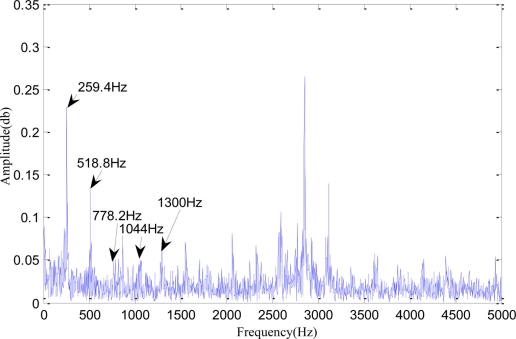
AE partial enlarged view of frequency spectra after redundant second generation wavelet denoising and Hilbert demodulation.

Figure 8 shows as AE partial enlarged view of frequency spectra after redundant second generation wavelet denoising and Hilbert demodulation. From Figure 8, it could be seen that the gear wheel of the epicyclic gearbox has a gear-mesh frequency of 259.4 Hz, a double frequency of 518.8 Hz, a triple frequency of 778.2 Hz, a quadruple frequency of 1,044 Hz, a quintuple frequency of 1300 Hz and high-order harmonics. Besides, the frequency spectra were more significant than those in the original image within 2,500 Hz.

From the perspective of mechanism analysis, we can see that if there is no abrasion, each gear has an involuted shape and the signal generated in the operation process is the single-frequency harmonic curve whose frequency is the gear mesh frequency; after the gear abrasion, the shape of gear changes and the signal generated in the operation process is the approximate periodic signal curve whose fundamental frequency is the gear mesh frequency; the more serious the abrasion is, the nearer time-domain curve gets to a square wave. So as the amplitude of mesh frequency (*i.e*., fundamental frequency) increases, the ultra-harmonics (double, triple, quadruple and quintuple fundamental frequency) amplitude of mesh frequency also significantly increases. The above analysis indicates that the gear wheel of the epicyclic gearbox may have suffered abrasion.

**Figure 9.**
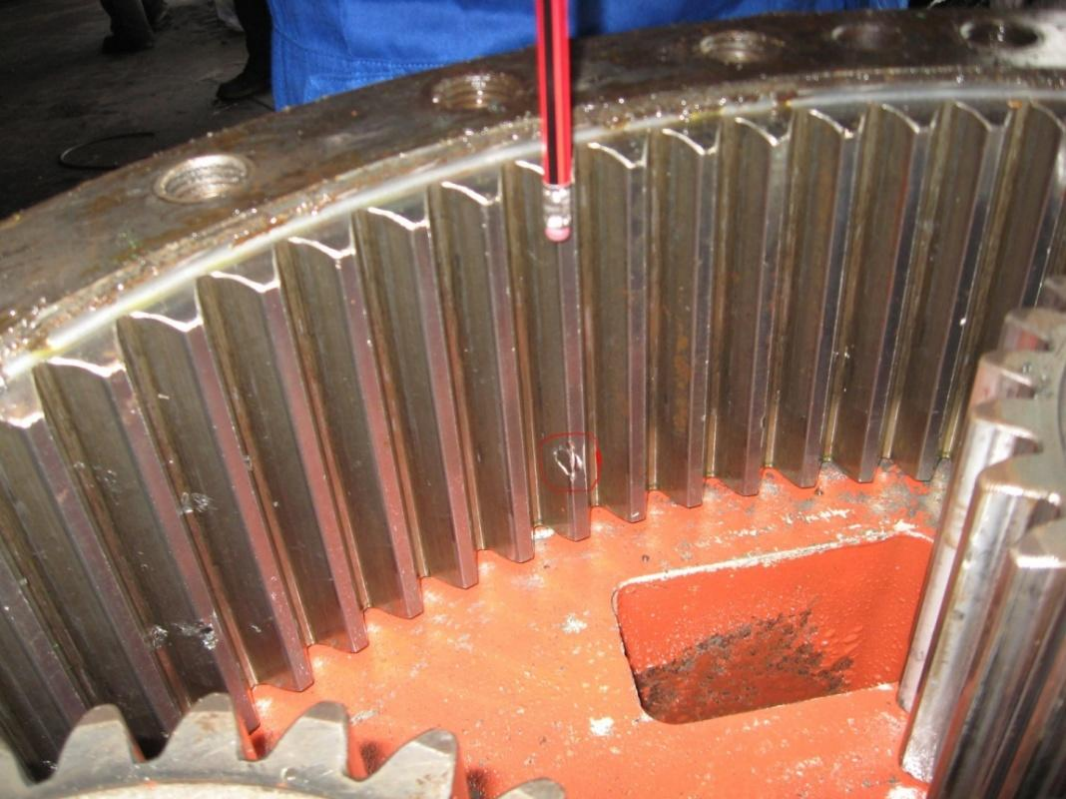
Picture of tooth-face abrasion and peel-off.

In the internal check on June 24, 2010, tooth-face abrasion and peel-off was detected on the gear wheel of the epicyclic gearbox (see Figure 9). The results of analysis and diagnosis matched the on-spot conditions.

**Figure 10.**
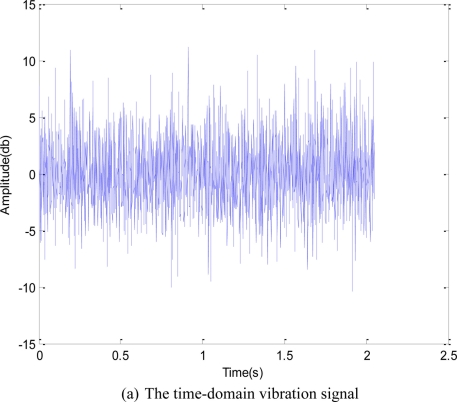

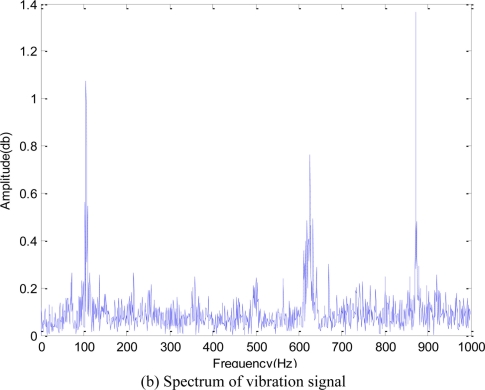
Vibration signals in time-domain and frequency-domain.

**Figure 11.**
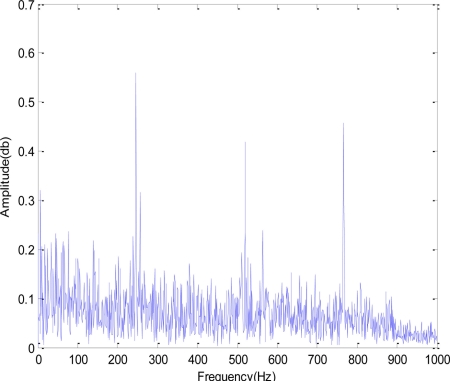
Spectrum of vibration signal after redundant second generation wavelet denoising and Hilbert demodulation.

The vibration sensors mounted on identical sites. Figure 10 shows the vibration signals in the time-domain and the frequency-domain, no significant characteristics. The method processing acoustic emission signals were also used to analyze the vibration signals (see Figure 11), also no significant characteristics. Therefore, the acoustic emission testing diagnosis was more effective than the vibration testing diagnosis in certain aspects.

## 5. Conclusions

The application of redundant second generation wavelet and acoustic emission testing in gear fault diagnosis was investigated in this study. Through the analysis of on-spot cases, the following conclusions were obtained:

1. The acoustic emission testing diagnosis could be applied to the fault diagnosis of on-spot low-speed heavy-duty gears and was a crucial supplement to the vibration testing diagnosis.
2. The length of the approximation signals and detail signals obtained after the decomposition of redundant second generation wavelet was the same as that of original ones, so the amount of information was twice that of the original. Such a characteristic of redundant second generation wavelet guaranteed that it could achieve an effect better than conventional wavelet and second generation one. With the denoising technique based on redundant second generation wavelet, all fault shock information could be preserved, which was unmatchable for other denoising techniques.
3. The validity of this algorithm of the redundant second generation wavelet transform during the processing and denoising of acoustic emission signals was verified.
